# Poison

**Published:** 1870-12

**Authors:** 


### POISON.

The instant a person is known to have swallowed poison by design or accident, give water to drink, cold or warm, as fast as possible, a gallon or more at a time, and as fast as vomited drink more; tepid water is best, as it opens the pores of the skin and promotes vomiting, and thus gives the speediest cure to the poisonous article. If pains begin to be felt in the bowels, it shows that part at least of the poison has passed downwards; then large and repeated injections of tepid water should be given, the object in both cases being to dilute the poison as quickly and as largely as possible. Do not wait for warm water—take that which is nearest at hand, cold or warm, for every second of time saved is of immense importance; at the same time send instantly for a physician, and as soon as he comes, turn the case into his hands, telling him what you have done. This simple fact cannot be too widely published; it is not meant to say that drinking a gallon or two of simple water will cure every case of poisoning, but it will cure many, and benefit all by its rapidly-diluting quality.

